# Effects of obesity on breast aromatase expression and systemic metabo-inflammation in women with *BRCA1* or *BRCA2* mutations

**DOI:** 10.1038/s41523-021-00226-8

**Published:** 2021-03-01

**Authors:** Neil M. Iyengar, Xi Kathy Zhou, Hillary Mendieta, Omar El-Hely, Dilip D. Giri, Lisle Winston, Domenick J. Falcone, Hanhan Wang, Lingsong Meng, Taehoon Ha, Michael Pollak, Monica Morrow, Andrew J. Dannenberg

**Affiliations:** 1 Departments of Medicine, Memorial Sloan Kettering Cancer Center, New York, NY USA; 2 Departments of Medicine, Weill Cornell Medical College, New York, NY USA; 3 Departments of Population Health Sciences, Weill Cornell Medical College, New York, NY USA; 4 Departments of Pathology, Memorial Sloan Kettering Cancer Center, New York, NY USA; 5 Departments of Pathology and Laboratory Medicine, Weill Cornell Medical College, New York, NY USA; 6 Departments of Medicine and Oncology, McGill University, Montreal, QC Canada; 7 Departments of Surgery, Memorial Sloan Kettering Cancer Center, New York, NY USA

**Keywords:** Breast cancer, Breast cancer

## Abstract

Obesity is associated with an increased risk of breast cancer in post-menopausal women and decreased risk in pre-menopausal women. Conversely, in *BRCA1/2* mutation carriers, pre-menopausal obesity is associated with early-onset breast cancer. Here we show that obese, pre-menopausal *BRCA1/2* mutation carriers have increased levels of aromatase and inflammation in the breast, as occurs in post-menopausal women. In a prospective cohort study of 141 women with germline *BRCA1* (*n* = 74) or *BRCA2* (*n* = 67) mutations, leptin, and aromatase expression were higher in the breast tissue of obese versus lean individuals (*P* < 0.05). Obesity was associated with breast white adipose tissue inflammation, which correlated with breast aromatase levels (*P* < 0.01). Circulating C-reactive protein, interleukin-6, and leptin positively correlated with body mass index and breast aromatase levels, whereas negative correlations were observed for adiponectin and sex hormone-binding globulin (*P* < 0.05). These findings could help explain the increased risk of early-onset breast cancer in obese *BRCA1/2* mutation carriers.

## Introduction

Mutant *BRCA1* and *BRCA2* DNA repair enzymes are causally linked to an increased risk of several malignancies including breast and ovarian cancers^1^. Not all women with mutations in *BRCA1* or *BRCA2* develop breast cancer and little is known about the factors that influence penetrance, making it difficult to personalize decisions concerning risk-reducing interventions. Previous epidemiological studies have suggested that obesity prior to menopause is associated with elevated risk and earlier onset of breast cancer in *BRCA* mutation carriers^2,3^. However, in unselected, predominantly wild-type populations, obesity is associated with a decreased risk of pre-menopausal breast cancer^4–8^. The mechanisms through which obesity contributes to early-onset breast cancer in *BRCA1/2* mutation carriers are incompletely understood.

It has been suggested that cancers occur at hormone-sensitive sites in *BRCA* mutation carriers owing at least in part to the pro-proliferative and mutagenic effects of estrogens^9–12^. The biosynthesis of estrogens is catalyzed by aromatase. Aromatase is expressed in breast adipose stromal cells and can be induced by both adipokines such as leptin and pro-inflammatory mediators including IL-6 and prostaglandin E_2_^13,14^. Previous studies that did not focus specifically on *BRCA1/2* mutation carriers demonstrated that levels of aromatase in the breast positively correlate with body mass index (BMI), breast adipocyte size, and white adipose tissue inflammation (WATi) manifested as crown-like structures of the breast (CLS-B)^15,16^. Post-menopausal status is independently associated with breast WATi, which contributes to elevated breast cancer risk in this population^16^. Systemic metabo-inflammation, characterized by altered levels of metabolic and inflammatory factors in the blood, may also play a role in the pathogenesis of breast cancer in the obese. For example, reduced levels of sex hormone-binding globulin (SHBG) have been observed in obese women leading to elevated levels of free estrogens in blood^17^. Pre- or post-menopausal women with impairments in DNA repair due to germline BRCA1/2 mutations may be more suceptible to the pro-mutagenic and pro-proliferative effects of free estrogens, which could lead to increased breast cancer risk.

Currently, women identified as *BRCA* mutation carriers are provided with few options to reduce their risk of developing breast cancer. Although prophylactic bilateral mastectomy and oophorectomy protect against breast cancer development^18^, many young women are reluctant to undergo risk-reducing surgery and opt for surveillance as a means to mitigate risk. Notably, pharmacologic therapy including tamoxifen and aromatase inhibitors may reduce the risk of breast cancer in *BRCA* mutation carriers, though these agents are associated with considerable toxicity^19,20^.

Despite evidence that estrogens are likely to play a role in the pathogenesis of breast cancer in *BRCA* mutation carriers, the effect of excess body fat on breast aromatase has not been examined in this high-risk population. Accordingly, a major objective of this study was to determine whether excess body fat or breast WATi are associated with increased aromatase expression in the breast microenvironment of mutation carriers. A second goal was to identify blood biomarkers that indicate elevated intra-breast aromatase levels.

## Results

### Excess body fat, breast adipocyte hypertrophy, and WATi are associated with higher breast expression of aromatase

In all, 141 women who underwent mastectomy for breast cancer treatment or prevention were included in the study: 74 were positive for *BRCA1* mutations and 67 had *BRCA2* mutations. Clinicopathological features are shown in Table 1. Patients with *BRCA1* and *BRCA2* mutations were of a similar age, BMI, and menopausal status. A greater percentage of hormone receptor-positive breast cancer was found in women with *BRCA2* than *BRCA1* mutations (44.78% vs. 22.97%). By contrast, triple-negative breast cancer was more common among women with *BRCA1* than *BRCA2* mutations (28.38% vs. 7.46%).

**Table 1:** Baseline characteristics of study population.

Variables	All (*n* = 141)	BRCA1 (*n* = 74)	BRCA2 (*n* = 67)	*P*
Age, median (IQR)	43 (37–50)	40 (33–50)	45 (38–50)	0.095
*BMI (%)*				
Normal/underweight	71 (50.35%)	43 (58.11%)	28 (41.79%)	
Overweight	40 (28.37%)	16 (21.62%)	24 (35.82%)	
Obese	30 (21.28%)	15 (20.27%)	15 (22.39%)	0.114
*WATi, n (%)*				
Non-inflamed	53 (37.59%)	30 (40.54%)	23 (34.33%)	
Inflamed	88 (62.41%)	44 (59.46%)	44 (65.67%)	0.489
*Race, n (%)*				
Asian	1 (0.71%)	0 (0%)	1 (1.49%)	
Black	10 (7.09%)	4 (5.41%)	6 (8.96%)	
White	113 (80.14%)	61 (82.43%)	52 (77.61%)	
American Indian/Alaska native	1 (0.71%)	1 (1.35%)	0 (0%)	
Other	5 (3.55%)	0 (0%)	5 (7.46%)	
Not reported	11 (7.8%)	8 (10.8%)	3 (4.48%)	0.042
*Menopausal status, n (%)*				
Pre	85 (60.71%)	44 (59.46%)	41 (62.12%)	
Post	55 (39.29%)	30 (40.54%)	25 (37.88%)	
Not reported	1 (0.71%)	0 (0%)	1 (1.49%)	0.728
*Tumor subtype, n (%)*				
HR+/HER2–	47 (33.33%)	17 (22.97%)	30 (44.78%)	
HER2+	3 (2.13%)	2 (2.7%)	1 (1.49%)	
Triple negative	26 (18.44%)	21 (28.38%)	5 (7.46%)	
Noninvasive or benign	65 (46.1%)	34 (45.95%)	31 (46.27%)	0.002

*IQR* interquartile range, *BMI* body mass index, *WATi* white adipose tissue inflammation, *HR* hormone receptor.

**Fig. 1:**
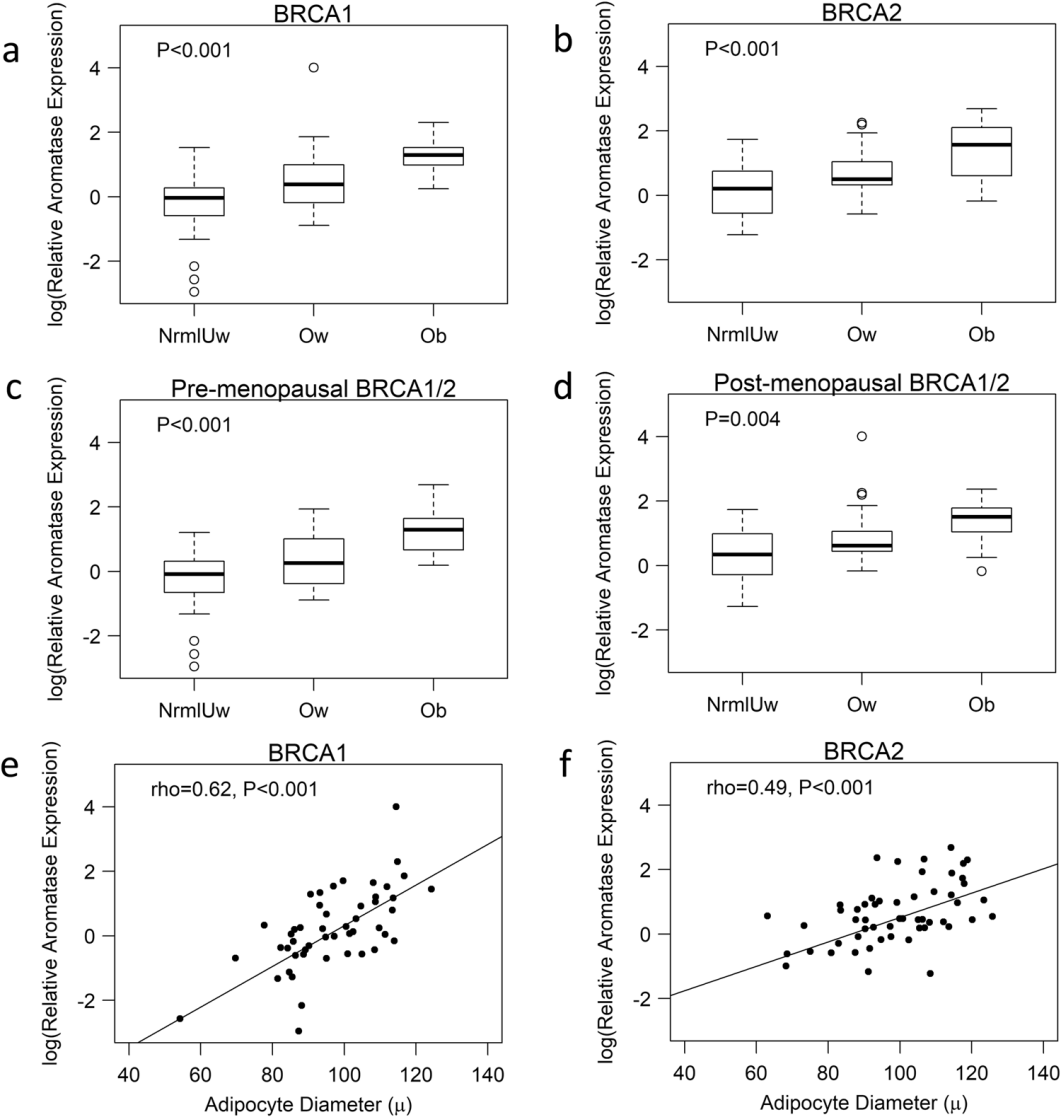
Effect of excess body fat on breast aromatase in women with *BRCA1* and *BRCA2* mutations. Box plots **a**–**d** showing: **a** Relative aromatase expression by BMI category in *BRCA1* mutation carriers. **b** Relative aromatase expression by BMI category in *BRCA2* mutation carriers. **c** Relative aromatase expression by BMI category in pre-menopausal *BRCA1/2* mutation carriers. **d** Relative aromatase expression by BMI category in post-menopausal *BRCA1/2* mutation carriers. Box boundaries represent upper and lower quartiles, centerline represents median, whiskers represent data points within 1.5× interquartile range from the box, and circles represent data points lying beyond the extremes of the whiskers. **e** Relative aromatase expression by adipocyte diameter in *BRCA1* mutation carriers. **f** Relative aromatase expression by adipocyte diameter in *BRCA2* mutation carriers.

**Fig. 2:**
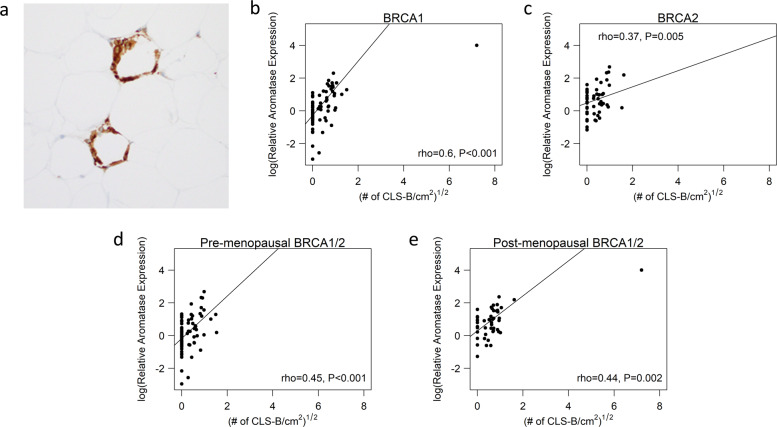
Breast WATi is associated with elevated levels of aromatase. **a** Anti-CD68 immunostaining showing CLS-B (×200). **b** Relative aromatase expression by the severity of white adipose tissue inflammation (CLS-B/cm^2^) in *BRCA1* mutation carriers. **c** Relative aromatase expression by severity of white adipose tissue inflammation (CLS-B/cm^2^) in *BRCA2* mutation carriers. **d** Relative aromatase expression by severity of white adipose tissue inflammation (CLS-B/cm^2^) in pre-menopausal *BRCA1/2* mutation carriers. **e** Relative aromatase expression by severity of white adipose tissue inflammation (CLS-B/cm^2^) in post-menopausal *BRCA1/2* mutation carriers.

**Fig. 3:**
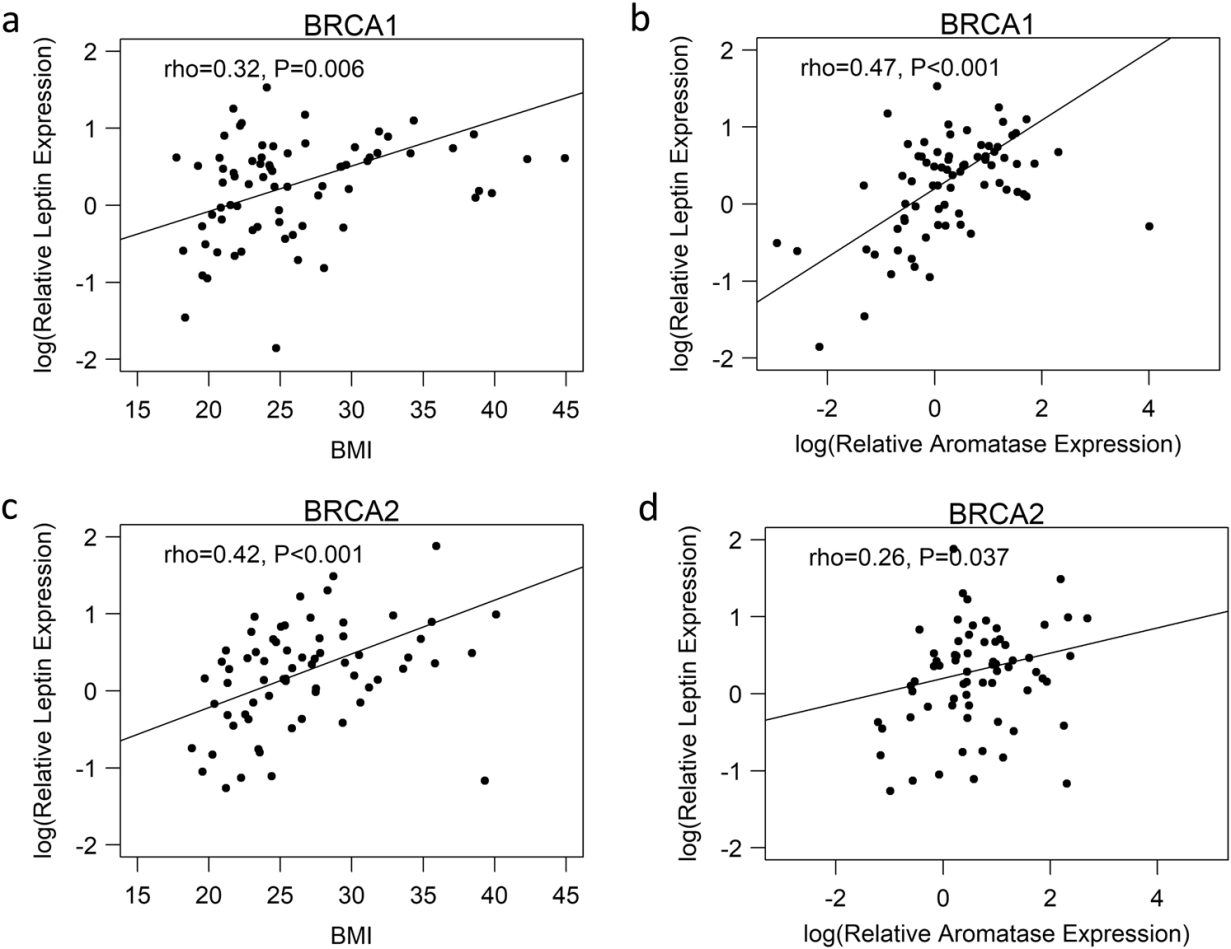
Breast leptin levels correlate with BMI and aromatase in women with *BRCA1* and *BRCA2* mutations. **a** Relative leptin expression by BMI (continuous) in *BRCA1* mutation carriers. **b** Relative leptin expression versus relative aromatase expression in *BRCA1* mutation carriers. **c** Relative leptin expression by BMI (continuous) in *BRCA2* mutation carriers. **d** Relative leptin expression versus relative aromatase expression in *BRCA2* mutation carriers.

Breast aromatase mRNA levels were higher in obese vs. lean women with *BRCA1* and *BRCA2* mutations (Fig. 1a, b). When stratified by menopausal status, aromatase levels were higher in obese vs. lean pre- and post-menopausal women (Fig. 1c, d). Given the link between excess body fat and increased aromatase expression, we also correlated breast adipocyte size with aromatase levels. As shown in Fig. 1e, f, a positive correlation was found between adipocyte diameter and aromatase expression in women with either *BRCA1* or *BRCA2* mutations. In previous studies that did not focus on heritable breast cancer, the severity of breast WATi, as defined by the density of CLS-B (Fig. 2a), was reported to correlate with both breast adipocyte size and aromatase expression^16,21^. Here, we demonstrate in both mutant *BRCA1* and *BRCA2* cases that aromatase levels correlated with the severity of breast WATi (Fig. 2b, c). When stratified by menopausal status, aromatase levels correlated with the severity of breast WATi in both pre- and post-menopausal women (Fig. 2d, e). Leptin, a pro-inflammatory adipokine that increases in association with obesity, can induce aromatase^22^. Hence, we investigated the relationship between BMI, leptin, and aromatase expression in the breast tissue of both *BRCA1* and *BRCA2* mutation carriers. Levels of leptin positively correlated with both BMI and aromatase (Fig. 3).

### Obesity, systemic factors, and breast aromatase

Circulating factors measured in fasted blood are presented in Table 2 and are stratified by BMI. Elevated BMI was associated with increased circulating levels of high-sensitivity C-reactive protein (hsCRP), IL-6, and leptin (*P* < 0.05) in women with either *BRCA1* or *BRCA2* mutations. In patients with *BRCA1* or *BRCA2* mutations, reduced levels of circulating adiponectin and SHBG were detected in association with elevated BMI (*P* < 0.05). Next, we correlated circulating factors with a range of breast measurements including the severity of WATi (#CLS-B/cm^2^), adipocyte diameter, and aromatase mRNA levels (Table 3). In women with either *BRCA1* or *BRCA2* mutations, breast WATi (# CLS-B/cm^2^) and adipocyte diameters correlated positively with blood levels of hsCRP, IL-6, and leptin, whereas an inverse relationship was detected for adiponectin and SHBG levels. Notably, blood levels of hsCRP, IL-6, leptin, and insulin positively correlated with breast aromatase mRNA levels in women with either *BRCA1* or *BRCA2* mutations (Table 3). In contrast, a negative correlation was observed for circulating levels of adiponectin and SHBG with breast aromatase mRNA levels (Table 3).

**Table 2:** Measured blood factors stratified by body mass index.

Factors^1^	BRCA1 cases			
	Uw/normal (*n* = 26)	Overweight (*n* = 17)	Obese (*n* = 12)	*P*^2^
hsCRP, ng/mL				
Median (IQR)	6.61 (6.14–7.39)	7.49 (6.36–8.32)	9.01 (7.84–9.56)	<0.001
IL-6, pg/mL				
Median (IQR)	0 (−0.2–0.53)	0.74 (0.04–1.52)	1.13 (0.96–1.81)	<0.001
Leptin, pg/mL				
Median (IQR)	9.02 (8.49–9.27)	9.52 (9.4–9.83)	10.35 (10.19–10.7)	<0.001
Adiponectin, mg/mL				
Median (IQR)	9.52 (9.21–9.79)	9.21 (8.81–9.76)	8.73 (8.44–9.34)	0.006
Insulin, mU/L				
Median (IQR)	1.57 (0.96–1.96)	1.4 (1.23–1.69)	1.94 (1.33–2.45)	0.213
SHBG, nmol/L				
Median (IQR)	4.35 (4.18–4.69)	4.12 (4.0–4.21)	3.62 (3.17–3.8)	<0.001

	BRCA2 cases			
	Uw/normal (*n* = 21)	Overweight (*n* = 18)	Obese (*n* = 10)	*P*^2^
hsCRP, ng/mL				
Median (IQR)	6.51 (5.59–6.9)	7.25 (6.17–7.77)	8.41 (8.06–8.66)	<0.001
IL-6, pg/mL				
Median (IQR)	−0.04 (−0.27–0.48)	0.8 (0.4–0.96)	0.86 (0.32–1.18)	0.015
Leptin, pg/mL				
Median (IQR)	9.19 (8.64–9.49)	9.86 (9.38–10.26)	10.31 (10.23–10.44)	<0.001
Adiponectin, mg/mL				
Median (IQR)	9.46 (9.22–9.68)	9.3 (9.07–9.43)	9.02 (8.9–9.35)	0.046
Insulin, mU/L				
Median (IQR)	1.18 (0.34–1.61)	1.68 (1.17–2.05)	1.45 (1.33–1.59)	0.082
SHBG, nmol/L				
Median (IQR)	4.42 (3.92–4.64)	4.09 (3.81–4.41)	3.7 (3.15–3.94)	0.013

^1^Log transformed.

^2^Based on the non-parametric Wilcoxon rank-sum test.

*IQR* interquartile range, *Uw* underweight, *hsCRP* high-sensitivity C-reactive protein, *IL-6* interleukin-6, *SHBG* sex hormone-binding globulin.

**Table 3:** Correlations among blood biomarkers, BMI, and breast endpoints.

Blood marker	BMI	BRCA1 cases		
		#CLS-B/cm^2^	Breast adipocyte diameter	Breast aromatase
hsCRP				
* ρ* (Spearman’s)	0.56	0.6	0.5	0.45
* P*	<0.001	<0.001	<0.001	0.002
IL-6				
* ρ* (Spearman’s)	0.53	0.48	0.28	0.36
* P*	<0.001	<0.001	0.059	0.013
Leptin				
* ρ* (Spearman’s)	0.76	0.46	0.45	0.54
* P*	<0.001	0.001	0.002	<0.001
Adiponectin				
* ρ* (Spearman’s)	−0.45	−0.5	−0.31	−0.48
* P*	<0.001	<0.001	0.035	<0.001
Insulin				
* ρ* (Spearman’s)	0.24	0.28	0.34	0.34
* P*	0.079	0.052	0.018	0.018
SHBG				
* ρ* (Spearman’s)	−0.56	−0.57	−0.39	−0.56
* P*	<0.001	<0.001	0.007	<0.001

		BRCA2 cases		
hsCRP				
* ρ* (Spearman’s)	0.67	0.39	0.46	0.38
* P*	<0.001	0.015	0.003	0.0099
IL-6				
* ρ* (Spearman’s)	0.4	0.37	0.47	0.52
* P*	0.005	0.021	0.002	<0.001
Leptin				
* ρ* (Spearman’s)	0.78	0.46	0.34	0.32
* P*	<0.001	0.004	0.029	0.029
Adiponectin				
* ρ* (Spearman’s)	−0.37	−0.32	−0.46	−0.46
* P*	0.008	0.052	0.003	0.001
Insulin				
* ρ* (Spearman’s)	0.35	0.12	0.26	0.29
* P*	0.013	0.462	0.101	0.051
SHBG				
* ρ* (Spearman’s)	−0.53	−0.38	−0.54	−0.42
* P*	<0.001	0.019	<0.001	0.004

## Discussion

The current study provides insights into the potential mechanisms by which excess body fat increases breast cancer penetrance in women with *BRCA1* or *BRCA2* mutations, including in pre-menopausal women. Although there is extensive literature suggesting that estrogens play a role in the pathogenesis of breast cancer in *BRCA1/2* mutation carriers, this study investigated the relationship between BMI and breast aromatase expression. Elevated BMI was associated with increased aromatase levels in both pre-menopausal and post-menopausal *BRCA1/2* mutation carriers. Consistent with prior studies that focused primarily on patients without a known genetic predisposition to breast cancer, the severity of WATi correlated with levels of aromatase in pre- and post-menopausal *BRCA* mutation carriers^15,21^. Notably, the increase in aromatase levels observed in association with breast WATi is likely to be functionally important because breast WATi has been associated with an increased ratio of estrogens to androgens in blood and breast adipose tissue^23^. Here we demonstrate that breast tissue and blood levels of leptin, a biomarker of adiposity and inducer of aromatase^22^, positively correlate with both BMI and aromatase expression in women with either *BRCA1* or *BRCA2* mutations. Collectively, these findings suggest that excess adiposity drives inflammation and local estrogen production, which ultimately promotes tumor growth in hyperadipose individuals with DNA repair insufficiency owing to germline *BRCA1 or BRCA2* mutations.

Our findings may help to explain why obesity increases the risk of early-onset breast cancer in *BRCA1/2* mutation carriers, including those who are pre-menopausal. In a seminal study published by King and colleagues, two modifiable risk factors were associated with breast cancer penetrance in *BRCA1/*2 mutation carriers—obesity and physical activity^3^. Specifically, obesity at menarche or age 21 and lack of physical activity during adolescence was associated with higher risk of early-onset breast cancer in *BRCA1/2* mutation carriers. Conversely, in non-carrier populations, obesity has been associated with lower risk of pre-menopausal breast cancer^4–8^. For example, in a pooled analysis of over 700,000 pre-menopausal women, BMI at ages 18–24 was inversely associated with breast cancer risk. The association was stronger for hormone receptor-positive tumors. In our study, we found that obese pre-menopausal women had higher aromatase levels and inflammation in the breast compared to lean individuals. Higher levels of aromatase and, consequently, free estrogens in the breast may compound breast cancer risk in patients who have defective DNA repair mechanisms. Notably, our study used BMI at the time of breast cancer diagnosis while the association between obesity and early-onset breast cancer in the study reported by King et al. used BMI at menarche. Nonetheless, BMI during puberty has been reported to predict adult obesity, suggesting a chronic state^24^.

Systemic factors related to altered metabo-inflammation may also modify the risk of breast cancer in those with excess body fat^25,26^. In fact, there is extensive literature supporting the importance of systemic factors in the pathogenesis of sporadic breast cancer^27,28^. Little is known, however, about this relationship in patients with a hereditary predisposition to breast cancer. In addition to evaluating the relationship between BMI and systemic factors, we also correlated levels of systemic factors with breast adipocyte size, WATi, and aromatase expression. Elevated BMI was associated with increased blood levels of hsCRP, IL-6, and leptin in women with either *BRCA1* or *BRCA2* mutations. In contrast, increased BMI was associated with reduced levels of both adiponectin, an anti-inflammatory adipokine, and SHBG. The reduction in SHBG would be anticipated to lead to increased free estradiol, which could also increase the risk of breast cancer, particularly in pre-menopausal mutation carriers^29^. Blood levels of hsCRP, IL-6, and leptin positively correlated with breast adipocyte diameter and the severity of breast WATi; levels of adiponectin and SHBG in serum negatively correlated with breast adipocyte size and the severity of breast WATi. Levels of circulating biomarkers of inflammation including hsCRP, IL-6, and leptin all positively correlated with breast aromatase levels, whereas an inverse correlation was observed for adiponectin and SHBG. Insulin levels also positively correlated with breast aromatase expression in women with either *BRCA1* or *BRCA2* mutations. Collectively, these changes in systemic factors in association with obesity resemble the changes typically observed in association with BMI in women without a genetic predisposition to breast cancer, and could increase the penetrance of breast cancer in *BRCA1* and *BRCA2* mutation carriers^30^.

To our knowledge, this is the largest study of breast aromatase and inflammation in a cohort of patients all with known *BRCA1/2* mutations. Additional strengths include stratification by BMI and menopausal status and analyses of multiple circulating factors that are involved in the pathogenesis of obesity-related cancer. However, our study did not include direct measurement of circulating or tissue levels of estrogens. Nonetheless, breast WATi is a surrogate of higher circulating estrogen:androgen ratio^23^. The association between BMI and lower levels of SHBG in our cohort is also consistent with higher circulating free estrogens. Increased estrone levels in breast tissue have been shown to promote inflammation, tumor-initiating stem cells, and the growth of hormone-sensitive breast tumors^31^. Higher levels of adipose tissue-derived pro-inflammatory estrogens in the setting of obesity and DNA repair deficit may thus lead to early-onset breast cancer in *BRCA1/2* mutation carriers. It is also possible that inflammation and higher insulin levels could lead to breast tumorigenesis via estrogen-independent mechanisms. In our cohort, we found an enrichment of triple-negative breast cancers in *BRCA1* mutation carriers and hormone receptor-positive cancers in *BRCA2* mutation carriers. However, we did not find an association between BMI and tumor subtype in pre-menopausal women in our cohort.

Overall, these findings provide a mechanism that includes interactions among excess body fat, inflammation, metabolic dysfunction, and aromatase in the breast that help to explain why excess body fat is likely to increase the penetrance of breast cancer in *BRCA1* and *BRCA2* mutation carriers. The findings are supportive of the use of strategies that decrease adiposity and lower estrogen levels to reduce the risk of breast cancer in these high-risk women. Although mastectomy is required to definitively reduce the risk of breast cancer, weight loss may be beneficial in those with excess body fat who choose to delay or defer undergoing surgery. Based on the current findings, it will be worthwhile to determine whether reducing weight will be associated with changes in blood biomarkers both because of their link to the pathogenesis of breast cancer and because they correlate with levels of intra-breast aromatase.

## Methods

### Study population and biospecimen acquisition

This study was approved by the Institutional Review Boards of Memorial Sloan Kettering Cancer Center (MSKCC) and Weill Cornell Medicine (New York, NY). Informed consent was provided by women undergoing mastectomy at MSKCC and breast WAT specimens were obtained under a standard tissue acquisition protocol. Patients underwent mastectomy for treatment of breast cancer or to reduce the risk of breast cancer. To ensure adequate tissue for analysis, patients undergoing lumpectomy were excluded. Clinicopathological data (age, race, *BRCA* mutation status, tumor subtype, and menopause status) were systematically extracted from the electronic medical record by research staff and physicians, and independent data review was carried out for quality assurance. Height and weight were prospectively recorded prior to surgery and used to calculate BMI. Standard definitions were used to categorize BMI as under- or normal weight (BMI < 25), overweight (BMI 25.0–29.9), or obese (BMI ≥ 30). Menopause status was described as either pre-menopausal or post-menopausal based on National Comprehensive Cancer Network (NCCN) criteria^32^. In brief, women were classified as post-menopausal if they had bilateral oophorectomy or reported permanent cessation of menses for 12 or more months in the absence of chemotherapy or endocrine therapy. All data were reviewed for accuracy independently by research staff and a physician.

For each subject, paraffin blocks and snap-frozen samples were prepared from breast WAT not involved by tumor on the day of mastectomy. Frozen samples were stored in the presence of RNAlater (Ambion). A 30 mL fasting blood sample was obtained preoperatively on the day of surgery. Blood was separated into serum and plasma by centrifugation within 3 h of collection and stored at −80 °C.

### WAT inflammation

Consistent with established methods, the presence or absence of breast WATi was determined by histologic assessment^15,16,33^. The presence of WATi was defined by CLS-B, which is comprised of a dead or dying adipocyte surrounded by CD68-positive macrophages^34^. Breast adipose tissue from the mastectomy specimen was formalin-fixed and paraffin-embedded (FFPE). Five FFPE blocks were prepared and one section per FFPE block (5 µm thick and ~2 cm in diameter) was generated such that five sections were stained for CD68, a macrophage marker (mouse monoclonal KP1 antibody; Dako; dilution 1:4000). Immunostained tissue sections were examined by the study pathologist (DG) using light microscopy to detect the presence or absence of CLS-B and record the number of CLS-B per slide. Digital photographs of each slide were generated and WAT area was measured with Image J Software (NIH, Bethesda, MD). The severity of WATi was quantified as the number of CLS-B per square centimeter of WAT (#CLS-B/cm^2^).

### Adipocyte measurement

Two Hematoxylin and Eosin (H&E) sections were generated from FFPE breast tissue in order to measure adipocyte diameters as previously described^10,11^. The H&E sections were photographed at ×20 using an Olympus BX50 microscope and MicroFire digital camera (Optronics). Mean diameters were calculated using measurements from 30 individual adipocytes for each patient using the linear dimensional tool in the Canvas 11 Software (ACD Systems International, Inc.).

### Quantitative PCR

Total RNA was isolated from human breast tissue using the RNeasy Mini Kit (Qiagen). RNA (2000 ng) was reverse transcribed using the qScript cDNA Synthesis Kit (QuantaBio), and the resulting cDNA used for real-time PCR amplification with Fast SYBR green PCR master mix on a 7500 HT real-time PCR system (Applied Biosystems). GAPDH was used as an endogenous normalization control. Commercial primers for LEP (QT00030261), and GAPDH (QT00079247) were purchased from Qiagen. In-house primers for aromatase (forward: 5′-CACATCCTCAATACCAGGTCC-3′ and reverse: 5′-CAGAGATCCAGACTCGCATG-3′) were synthesized by Sigma-Aldrich. Relative fold-induction was determined using the ΔΔC_T_analysis protocol.

### Blood assays

Plasma levels of leptin, adiponectin, hsCRP, SHBG, interleukin-6 (IL-6; R&D Systems, Minneapolis, MN), and insulin (Mercodia, Uppsala, Sweden), were measured by enzyme-linked immunosorbent assay.

### Biostatistical analyses

To examine relationships between two continuous variables, the Spearman correlation was used. Differences in a continuous variable across multiple categories were examined using the non-parametric Kruskal–Wallis test. Levels of a circulating factor and levels of relative expression of a gene were log-transformed for improving clarity in data visualization. For all analyses, statistical significance was set at two-tailed *P* < 0.05. All statistical analyses were conducted using R software (R Foundation for Statistical Computing, Vienna, Austria).

### Reporting summary

Further information on experimental design is available in the Nature Research Reporting Summary linked to this paper.

## Supplementary information

Reporting Summary Checklist

## Data Availability

The data sets that support the findings of this study are not publicly available in order to protect patient privacy. Data will be made available to authorized researchers who have received approval from the Memorial Sloan Kettering Cancer Center (MSKCC) Institutional Review Board. The data generated and analyzed during this study are described in the following metadata record: 10.6084/m9.figshare.13537076^35^.
