# Supplementary material for: Effects of obesity on breast aromatase expression and systemic metabo-inflammation in women with BRCA1 or BRCA2 mutations
Source: NPJ Breast Cancer. 2021 Mar 1;7:18. doi: 10.1038/s41523-021-00226-8 (PMC7921427; doi:10.1038/s41523-021-00226-8)
Supplement: Supplementary file 1 — Reporting Summary Checklist [file 41523_2021_226_MOESM1_ESM.pdf]

nature research

Corresponding author(s): Andrew Dannenberg
Last updated by author(s): Jan 8, 2021

Reporting Summary

Nature Research wishes to improve the reproducibility of the work that we publish. This form provides structure for consistency and transparency in reporting. For further information on Nature Research policies, see our Editorial Policies and the Editorial Policy Checklist.

Statistics

For all statistical analyses, confirm that the following items are present in the figure legend, table legend, main text, or Methods section.

n/a	Confirmed	
☐	☒	The exact sample size (*n*) for each experimental group/condition, given as a discrete number and unit of measurement
☐	☒	A statement on whether measurements were taken from distinct samples or whether the same sample was measured repeatedly
☐	☒	The statistical test(s) used AND whether they are one- or two-sided<br>*Only common tests should be described solely by name; describe more complex techniques in the Methods section.*
☐	☒	A description of all covariates tested
☐	☒	A description of any assumptions or corrections, such as tests of normality and adjustment for multiple comparisons
☐	☒	A full description of the statistical parameters including central tendency (e.g. means) or other basic estimates (e.g. regression coefficient) AND variation (e.g. standard deviation) or associated estimates of uncertainty (e.g. confidence intervals)
☒	☐	For null hypothesis testing, the test statistic (e.g. *F*, *t*, *r*) with confidence intervals, effect sizes, degrees of freedom and *P* value noted<br>*Give P values as exact values whenever suitable.*
☒	☐	For Bayesian analysis, information on the choice of priors and Markov chain Monte Carlo settings
☒	☐	For hierarchical and complex designs, identification of the appropriate level for tests and full reporting of outcomes
☒	☐	Estimates of effect sizes (e.g. Cohen's *d*, Pearson's *r*), indicating how they were calculated

Our web collection on statistics for biologists contains articles on many of the points above.

Software and code

Policy information about availability of computer code

Data collection	Data abstracted from medical records were collected and stored using Microsoft Excel software. Adipose tissue area was measured using NIH Image J software. Canvas 11 software from ACD Systems International was used to quantify adipocyte diameters.
Data analysis	All statistical analyses were conducted using the statistical computing language and environment R (https://www.r-project.org/).

For manuscripts utilizing custom algorithms or software that are central to the research but not yet described in published literature, software must be made available to editors and reviewers. We strongly encourage code deposition in a community repository (e.g. GitHub). See the Nature Research guidelines for submitting code & software for further information.

Data

Policy information about availability of data

All manuscripts must include a data availability statement. This statement should provide the following information, where applicable:
- Accession codes, unique identifiers, or web links for publicly available datasets
- A list of figures that have associated raw data
- A description of any restrictions on data availability

The datasets that support the findings of this study are not publicly available in order to protect patient privacy. Data will be made available to authorized researchers who have received approval from the Memorial Sloan Kettering Cancer Center (MSKCC) Institutional Review Board. Please contact Dr. Neil Iyengar, email address: iyengarn@mskcc.org, with data access requests. The data generated and analysed during this study are described in the following metadata record: https://doi.org/10.6084/m9.figshare.13537076.

Field-specific reporting

Please select the one below that is the best fit for your research. If you are not sure, read the appropriate sections before making your selection.

☒ Life sciences ☐ Behavioural & social sciences ☐ Ecological, evolutionary & environmental sciences

For a reference copy of the document with all sections, see nature.com/documents/nr-reporting-summary-flat.pdf

Life sciences study design

All studies must disclose on these points even when the disclosure is negative.

Sample size	We did not predetermine sample size in this cross-sectional study. We used all available samples collected under a biospecimen acquisition protocol from May 5, 2010 through September 4, 2018.
Data exclusions	No data were excluded from the analyses.
Replication	Findings from immunohistochemistry for adipose inflammation, adipocyte measurements aromatase PCR, and circulating biomarkers are consistent with prior reports by our group and others in BRCA wild type populations. Consistent with the current findings, we've previously shown that obesity and breast white adipose inflammation are associated with elevated levels of aromatase in cohorts of women who were primarily wild-type for BRCA1/2. Moreover, RNA-seq analysis has confirmed the aromatase qPCR findings
Randomization	This is a cross-sectional study, and randomization is not applicable.
Blinding	This is a cross-sectional study, and blinding is not applicable. Nonetheless, the study pathologist was blinded to the clinical data.

Reporting for specific materials, systems and methods

We require information from authors about some types of materials, experimental systems and methods used in many studies. Here, indicate whether each material, system or method listed is relevant to your study. If you are not sure if a list item applies to your research, read the appropriate section before selecting a response.

Materials & experimental systems

n/a	Involved in the study	
☐	☒	Antibodies
☒	☐	Eukaryotic cell lines
☒	☐	Palaeontology and archaeology
☒	☐	Animals and other organisms
☐	☒	Human research participants
☐	☒	Clinical data
☒	☐	Dual use research of concern

Methods

n/a	Involved in the study	
☒	☐	ChIP-seq
☒	☐	Flow cytometry
☒	☐	MRI-based neuroimaging

Antibodies

Antibodies used	Anti-CD68 antibody (mouse monoclonal KP1 antibody; Dako; dilution 1:4,000)
Validation	Optimized for immunohistochemistry (IHC) with validated protocols per manufacturer's website: https://www.agilent.com/en/product/immunohistochemistry/antibodies-controls/primary-antibodies/cd68-(concentrate)-76550

Human research participants

Policy information about studies involving human research participants

Population characteristics	This study included pre- and post-menopausal women with germline BRCA1 or BRCA2 mutations who underwent mastectomy for the treatment or prevention of breast cancer at Memorial Sloan Kettering Cancer Center (median age 43, range 37 to 50 years).
Recruitment	All patients undergoing surgery at MSKCC are offered participation in the institutional biospecimen collection protocol that was used for this study.
Ethics oversight	This study was approved by the institutional review boards at Memorial Sloan Kettering Cancer Center and Weill Cornell Medicine.

Note that full information on the approval of the study protocol must also be provided in the manuscript.

Clinical data

Policy information about clinical studies

All manuscripts should comply with the ICMJE guidelines for publication of clinical research and a completed CONSORT checklist must be included with all submissions.

Clinical trial registration	N/A
Study protocol	Samples for this study were collected via a standard biospecimen collection protocol; details are provided in the methods section of the manuscript
Data collection	All data were collected at MSKCC and included patients who underwent breast surgery from May 5, 2010 through September 4, 2018.
Outcomes	Pre-defined outcomes included adipose inflammation, aromatase expression, and circulating biomarkers.
